# Comparing post-acute rehabilitation use, length of stay, and outcomes experienced by Medicare fee-for-service and Medicare Advantage beneficiaries with hip fracture in the United States: A secondary analysis of administrative data

**DOI:** 10.1371/journal.pmed.1002592

**Published:** 2018-06-26

**Authors:** Amit Kumar, Momotazur Rahman, Amal N. Trivedi, Linda Resnik, Pedro Gozalo, Vincent Mor

**Affiliations:** 1 Center for Gerontology and Health Care Research, Department of Health Services, Policy, and Practice, School of Public Health, Brown University, Providence, Rhode Island, United States of America; 2 Providence Veterans Affairs Medical Center, Providence, Rhode Island, United States of America; Edinburgh University, UNITED KINGDOM

## Abstract

**Background:**

Medicare Advantage (MA) and Medicare fee-for-service (FFS) plans have different financial incentives. Medicare pays predetermined rates per beneficiary to MA plans for providing care throughout the year, while providers serving FFS patients are reimbursed per utilization event. It is unknown how these incentives affect post-acute care in skilled nursing facilities (SNFs). The objective of this study was to examine differences in rehabilitation service use, length of stay, and outcomes for patients following hip fracture between FFS and MA enrollees.

**Methods and findings:**

This was a retrospective cohort study to examine differences in health service utilization and outcomes between FFS and MA patients in SNFs following hip fracture hospitalization during the period January 1, 2011, to June 30, 2015, and followed up until December 31, 2015. We linked the Master Beneficiary Summary File, Medicare Provider and Analysis Review data, Healthcare Effectiveness Data and Information Set data, the Minimum Data Set, and the American Community Survey. The 6 primary outcomes of interest in this study included 2 process measures and 4 patient-centered outcomes. Process measures included length of stay in the SNF and average rehabilitation therapy minutes (physical and occupational therapy) received per day. Patient-centered outcomes included 30-day hospital readmission, changes in functional status as measured by the 28-point late loss MDS-ADL scale, likelihood of becoming a long-term resident, and successful discharge to the community. Successful discharge from a SNF was defined as being discharged to the community within 100 days of SNF admission and remaining alive in the community without being institutionalized in any acute or post-acute setting for at least 30 days. We analyzed 211,296 FFS and 75,554 MA patients with hip fracture admitted directly to a SNF following an index hospitalization who had not been in a nursing facility or hospital in the preceding year. We used inverse probability of treatment weighting (IPTW) and nursing facility fixed effects regression models to compare treatments and outcomes between MA and FFS patients. MA patients were younger and less cognitively impaired upon SNF admission than FFS patients. After applying IPTW, demographic and clinical characteristics of MA patients were comparable with those of FFS patients. After adjusting for risk factors using IPTW-weighted fixed effects regression models, MA patients spent 5.1 (95% CI -5.4 to -4.8) fewer days in the SNF and received 463 (95% CI to -483.2 to -442.4) fewer minutes of total rehabilitation therapy during the first 40 days following SNF admission, i.e., 12.1 (95% CI -12.7 to -11.4) fewer minutes of rehabilitation therapy per day compared to FFS patients. In addition, MA patients had a 1.2 percentage point (95% CI -1.5 to -1.1) lower 30-day readmission rate, 0.6 percentage point (95% CI -0.8 to -0.3) lower rate of becoming a long-stay resident, and a 3.2 percentage point (95% CI 2.7 to 3.7) higher rate of successful discharge to the community compared to FFS patients. The major limitation of this study was that we only adjusted for observed differences to address selection bias between FFS and MA patients with hip fracture. Therefore, results may not be generalizable to other conditions requiring extensive rehabilitation.

**Conclusions:**

Compared to FFS patients, MA patients had a shorter course of rehabilitation but were more likely to be discharged to the community successfully and were less likely to experience a 30-day hospital readmission. Longer lengths of stay may not translate into better outcomes in the case of hip fracture patients in SNFs.

## Abstract 2

In a retrospective analysis of Medicare data, Vincent Mor and colleagues compare outcomes for patients with hip fracture under different models of care.

## Author summary

**Why was this study done?:**

- Enrollment in Medicare Advantage plans has increased significantly, from 5.3 million in 2003 to 19 million in 2017.
- Research suggests that the different incentives under which Medicare Advantage and Medicare fee-for-service operate are related to less medical service use by Medicare Advantage patients.
- No studies to date have compared the treatment and outcomes of Medicare Advantage and Medicare fee-for-service patients using post-acute skilled nursing facilities.

**What did the researchers do and find?:**

- We compared the treatment processes and outcomes experienced by 211,296 Medicare fee-for-service and 75,554 Medicare Advantage patients with hip fracture admitted to skilled nursing facilities directly from the hospital.
- Despite shorter length of skilled nursing facility stay and less rehabilitation therapy, Medicare Advantage patients had significantly lower hospital readmission rates and higher rates of successful community discharge than did fee-for-service patients.

**What do these findings mean?:**

- The findings of this study suggest that reducing the duration of rehabilitation care in skilled nursing facilities may be achieved without adversely affecting the quality of rehabilitation care or the health outcomes experienced by hip fracture patients.
- These findings cannot necessarily be extrapolated to other conditions requiring extensive rehabilitation, so future research should be undertaken to determine whether these findings can be extended beyond outcomes for hip fracture patients.
- Nonetheless, the results of this study could inform the current debate on value-based payment reform in post-acute care settings in the US.

## Introduction

In the United States, Medicare provides health insurance coverage to older adults through the traditional Medicare fee-for-service (FFS) program or the Medicare Advantage (MA) program. The FFS program gives an incentive for providers to provide more treatments because reimbursement is contingent on the amount of care. However, the MA program serves Medicare beneficiaries through private health insurance plans, which are paid a capitated amount to coordinate care via a more limited provider network than the traditional FFS program [1]. MA enrollment has grown from 6.8 million (16%) in 2006 to 19.0 million (33%) in 2017 [2,3], accompanied by large increases in post-acute care utilization by MA beneficiaries, especially in skilled nursing facilities (SNFs) [4]. Unfortunately, there is limited evidence on the utilization of SNF-based rehabilitation services by beneficiaries in MA plans or on how treatment and associated outcomes compare with those experienced by FFS beneficiaries.

Rehabilitation services, including physical therapy (PT) and occupational therapy (OT), play a critical role in preventing deconditioning, restoring functional status, and facilitating discharge to the community [5]. Resource utilization groups (RUGs) were introduced as the way per diem prospective payments are set for Medicare-reimbursed SNF care, classifying patients into reimbursement categories based on rehabilitation service use: ultra (720+ minutes/week), very high (500–719), high (325–499), medium (150–324), and low (45–149). Therefore, SNFs are incentivized to provide more OT and PT services to FFS patients to qualify for higher payments. Furthermore, as a per diem system, longer lengths of stay generate more revenue. Indeed, the Centers for Medicare and Medicaid Services (CMS) has reported that FFS patients’ therapy use has increased without significant changes in the acuteness of the conditions of newly admitted SNF patients [6].

MA plans must offer equal benefits during the SNF stay but do not necessarily use a RUG-based payment system nor even per diem prices. Since MA plans receive capitated per member per month payments, they are incentivized to limit the days of care and the intensity of therapy services, possibly leading to less therapy and earlier discharge, which may compromise outcomes. To date, however, there is limited literature documenting the impact of the different incentives under the MA and FFS programs in terms of SNF patients’ outcomes. One reason for the paucity of research on this issue has been limited access to utilization data on MA patients. In this paper, we take advantage of the mandatory Minimum Data Set (MDS) assessments completed on all admissions to nursing homes irrespective of their insurance status, as well as the availability of Medicare Provider and Analysis Review (MedPAR) hospital claims for over 90% of all MA members from hospitals receiving disproportionate share hospital (DSH) payments or educational training payments or supplements [7]. Having comparable data for both FFS and MA beneficiaries allowed us to compare the intensity, duration, and outcomes of rehabilitation therapy received by patients with hip fracture discharged from hospitals to SNFs. We selected patients with a hip fracture because it is prevalent and associated with adverse outcomes such as functional impairment, readmission, permanent institutionalization, and mortality and because most patients with hip fracture are discharged to SNFs [8–11]. The objective of this study was to compare SNF rehabilitation use, length of stay, and patient outcomes between FFS and MA enrollees discharged from hospitals to SNFs following hip fracture. We hypothesized that MA patients would receive less rehabilitation care and would therefore experience worse outcomes.

## Methods

### Study design and sample

**Fig 1:**
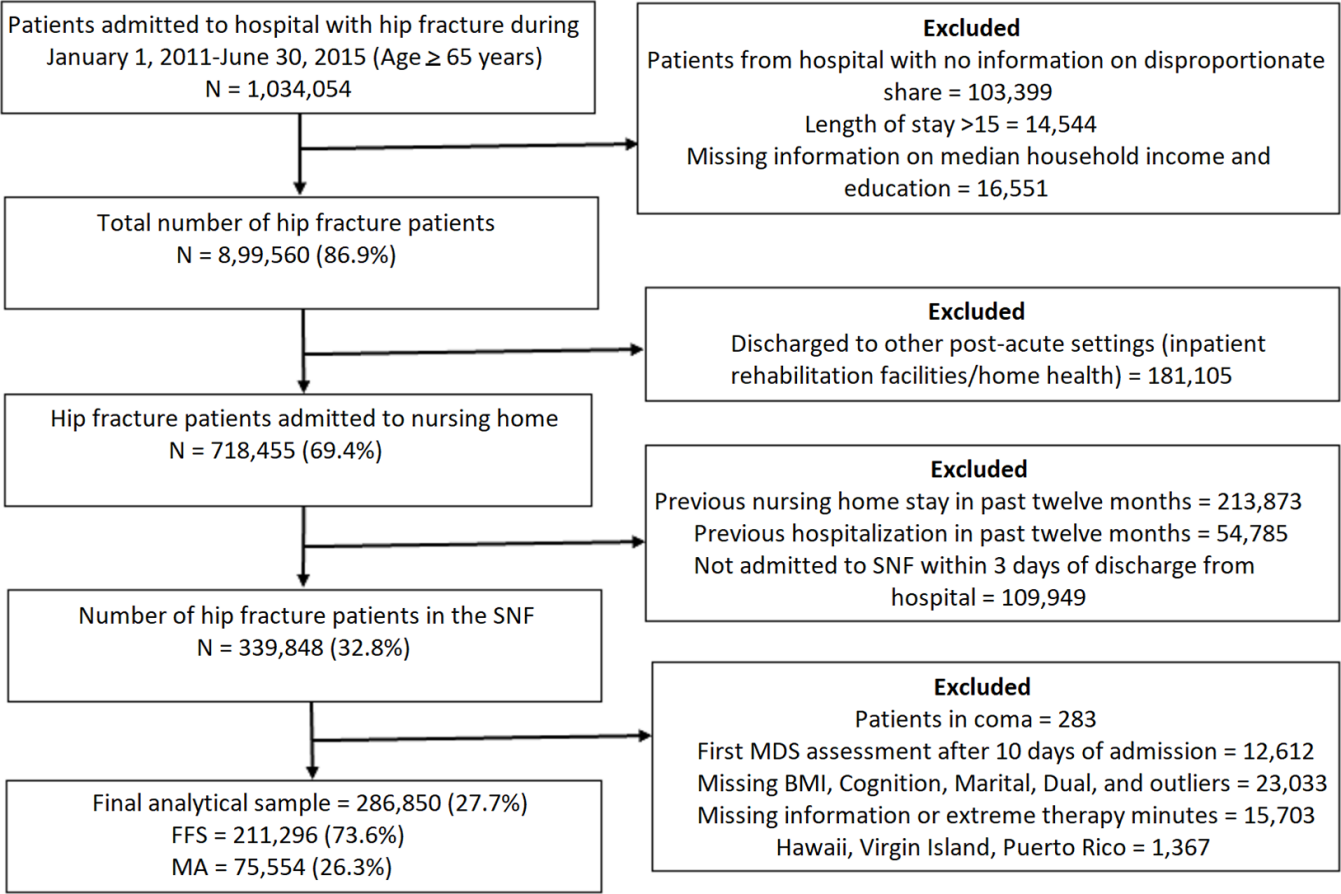
The derivation of the study sample. Dual, Medicare and Medicaid dual eligibility; MDS, Minimum Data Set; SNF, skilled nursing facility.

This was a retrospective cohort study seeking to examine the differences in health service utilization and outcomes between FFS and MA patients admitted in SNFs following hip fracture hospitalization. We selected patients admitted to a SNF following an index acute hospitalization who had not been in a nursing home or in a hospital in the preceding year and who were admitted directly to a nursing facility as indicated by the presence of an MDS admission record dated within 3 days of hospital discharge. Hip fracture patients were identified using Medicare Severity Diagnosis Related Group (MS-DRG) codes (533, 534, 535, 536) and primary ICD-9-CM diagnostic codes (82000, 82021, 82022, 82023, 82024, 82026, 82027, 82028, 82029, 82030, 82031, 82032, 8208, 82080, 82009, 8080) from the MedPAR files. Fig 1 summarizes the selection process, which yielded the final analytic sample of 286,850 hip fracture patients, 211,296 (74%) FFS and 75,554 (26%) MA patients, admitted to a SNF from the hospital during the period January 1, 2011, through June 30, 2015.

### Data sources

We used data from January 1, 2011, to December 31, 2015, from the Master Beneficiary Summary File (MBSF), MedPAR data, Healthcare Effectiveness Data and Information Set (HEDIS) data, the MDS (version 3.0), and the American Community Survey. Each data source is briefly described below. The MBSF includes demographic characteristics and monthly FFS or MA enrollment status. The MedPAR file contains claims for inpatient stays and includes information about diagnoses, surgical procedures, and hospital length of stay from hospitals participating in the inpatient prospective payment system. Since 2008, the MedPAR file includes claims from MA enrollees receiving care in hospitals receiving either DSH payments or Indirect Medical Education or Direct Graduate Medical Education adjustments, which account for over 90% of all MA hospitalizations [4]. A majority of safety-net hospitals in the US receive subsidies from Medicaid and Medicare (DSH payments, Medicaid Upper Payment Limit payments, Indirect Medical Education payments) and state/local indigent health programs because they serve traditionally low-income and marginalized populations. Our method of capturing hospital claims of MA patients was consistent with a recent paper by Huckfeldt et al. [4]. In addition, we linked MedPAR claims with HEDIS files to capture readmissions in non-DSH hospitals for MA patients. Thus, our study cohort was restricted to patients discharged from DSH hospitals.

HEDIS assembles information from medical records and administrative data on Medicare beneficiaries enrolled in MA plans and includes beneficiary-level information on rehospitalization within 30 days of original hospital discharge [12]. MDS assessments are mandatory for all admissions and residents of Medicare/Medicaid-certified nursing homes and are completed upon admission, upon discharge, and quarterly for long-term residents. The MDS assessment includes summary measures of cognitive and physical functioning, pain, and medication use, and detailed data on rehabilitation service utilization (minutes of PT and OT).

### Outcomes

The 6 primary outcomes of interest in this study included 2 process measures and 4 patient-centered outcomes. Process measures included length of stay in the SNF and the total number of OT and PT minutes. Patient outcomes included 30-day hospital readmission, change in functional status as measured by the 28-point late loss MDS-ADL scale, becoming a long-term resident, and successful discharge to the community.

SNF length of stay was defined as calendar days from admission to discharge based upon the MDS admission and discharge records. Since we included patients admitted to SNFs until June 30, 2015, and followed up until December 31, 2015, we kept the follow-up period uniform across all patients; therefore, we followed patients for up to 180 days in the SNF. Number of minutes of rehabilitation service received was calculated by using the total number of individual therapy minutes, concurrent minutes, and group minutes for PT and OT services across MDS assessment records up until day 40 of the patient’s stay. Since most stays are for less than 40 days, we calculated minutes of therapy received in the first 40 days of SNF stays and the average number of minutes per day by dividing the total number of therapy minutes from the SNF length of stay up to 40 days. We focused on rehabilitation therapy in the first 40 days because clinical trial evidence suggests early rehabilitation is important for optimizing functional recovery after hip fracture in older patients [13,14].

We examined all-cause readmission to the hospital within 30 days following discharge from the index hospitalization. Because MA enrollees might be readmitted to a non-DSH hospital that may not submit claims to Medicare, we merged the MedPAR and HEDIS files to estimate the 30-day hospital readmission rate for MA enrollees. We also examined whether SNF patients became long-stay nursing home residents, defined as remaining in a nursing home for more than 100 days. Recently, CMS proposed successful discharge to the community as one of the quality measures for SNFs [15–17]. Successful discharge from a SNF is defined as being discharged to the community within 100 days of SNF admission and remaining alive in the community without being institutionalized in any acute or post-acute setting for at least 30 days. We linked MDS with MedPAR and HEDIS files to construct the successful discharge measure. Functional status was measured using the validated MDS-ADL scale, which aggregates 7 activities of daily living (ADL) items into a 28-point scale with 0 indicating independence and 28 indicating total dependence [18,19]. Functional change was computed by subtracting the admission from the discharge score; scores were reversed from negative to positive to improve understanding. The higher the score, the greater the improvement.

### Sociodemographic and clinical characteristics

Sociodemographic characteristics included age, sex, race/ethnicity, marital status, and Medicare and Medicaid dual eligibility. These variables were extracted from the MBSF file. We obtained median household income and household educational information for the patient’s zip code of residence from the American Community Survey data [20]. We obtained the following patient characteristics from the inpatient MedPAR file: hospital length of stay, days in an intensive care unit, Hierarchical Condition Category (HCC), hospital-acquired conditions, and fracture management using ICD-9 procedure codes. The HCC is a risk adjustment measure developed by CMS for reimbursement of MA patients, but we applied the algorithm to capture comorbidities from MedPAR inpatient claims derived from the index hospitalization [21,22]. We used the following patient characteristics drawn from the admission MDS assessment: admission MDS-ADL score, pain, BMI, and cognition, measured using the Cognitive Function Scale, a validated measure derived from the MDS [23].

### Hospital and SNF characteristics

Safety-net hospitals in the US include both public and private hospitals that provide healthcare services to individuals regardless of their insurance status, including uninsured, Medicaid, and other vulnerable populations. Safety-net status of the hospital was determined according to the CMS DSH index. Hospitals in the highest quartile of the DSH index were defined as a safety-net hospitals [24]. SNF characteristics were obtained from the CMS’s Online Survey Certification and Reporting System (OSCAR), which reports staffing and facility characteristics. We included ownership (for profit versus non-profit) and whether the facility was part of a chain or not.

### Statistical analyses

To account for differences between the MA and FFS populations attributable to selection bias, we computed the inverse probability of treatment weighting (IPTW) as a propensity for being an MA enrollee based on observable characteristics. The propensity score estimates the probability of our hip fracture discharges being enrolled in an MA plan. Propensity was modeled as a function of all observable patient-level, zip-code-level, and state-level fixed effects. Patient-level characteristics included age, sex, race/ethnicity, marital status, length of stay in the hospital, number of days in the intensive care unit, fracture treatment, number of comorbidities, admission cognition, body mass index, hospital-acquired complications, HCC, and dual Medicare/Medicaid status. At the zip code level, we included patient’s residential zip code median household income and household education.

Following the calculation of the IPTW, we verified that the IPTW-weighted samples were balanced on patient characteristics across the 2 MA and FFS insurance groups (S1 Fig). We examined the difference in outcomes between MA and FFS using a linear probability model after applying the IPTW. We used linear probability models because the coefficient of MA indicator (with FFS as the reference category) provides a direct estimate of the risk-adjusted MA differential effect on the outcome while controlling for SNF facility-level unmeasured confounders. We also estimated logistic regression models to examine the binary outcome variables: 30-day hospital readmission, becoming a long-stay resident, and successful discharge to the community.

A recent study documented that MA patients are more likely to be discharged to SNFs with lower publicly reported star ratings according to the CMS’s Five-Star Quality Rating System [25]. Therefore, our final analyses using linear probability models compared all outcomes between FFS and MA patients using IPTW-weighted and SNF fixed effects, which compare the outcomes of MA and FFS beneficiaries within the same SNF facility to better account for unmeasured facility-level confounders. We did not estimate logistic regression models using SNF fixed effects because they assume a single positive outcome within a cluster (in our case SNF), and, in our analytic setup, any patient within a SNF can have a positive outcome. In all our regression models, standard errors were adjusted for clustering at the SNF level.

Approximately 3.5% of the patients in our analytical cohort had severe cognitive impairment, which may influence care processes and patients’ outcomes. Therefore, we conducted sensitivity analyses after excluding patients with severe cognitive impairment (S2 and S3 Tables). In addition, to rule out favorable risk selection and to make sure that our IPTW was working adequately, we performed sensitivity analyses comparing all-cause 6-month and 1-year mortality rates between MA and FFS patients. All analyses were prespecified except the addition of sensitivity analyses comparing process of care and patient outcomes after excluding patients with severe cognitive impairment.

### Ethics and resource sharing statement

The study was reviewed and approved by the Institutional Review Board of Brown University with a waiver of consent for the use of secondary identifiable data. This study is reported as per the STROBE guidelines (S1 STROBE Checklist). These person-level data (i.e., MDS, MBSF, MedPAR, and HEDIS) are covered under the strict terms of a data use agreement with CMS. Interested researchers may replicate the study using the data in the paper and its Supporting Information files. The authors requested use of the data from CMS through data use agreement #18900. To replicate the study requires using 100% Medicare inpatient claims data from the MedPAR extract files, MDS (version 3.0), and HEDIS data. ResDAC provides technical assistance to researchers interested using CMS Medicare and Medicaid data (https://www.resdac.org/about-resdac/our-services). We have deposited specific information regarding the cohort selection approach, variable definitions, and outcome specifications constructed across different datasets in an electronic repository as a guide for invesigators who obtain the requisite data use agreement for the relevant data sources and want to replicate our study (https://repository.library.brown.edu/studio/item/bdr:786344/).

## Results

The mean age of hip fracture patients discharged to SNFs was 83.9 years (SD 7.5), and the sample was predominantly female (77.2%) and white (90.2%). Given the large sample size, comparisons of the characteristics of MA and FFS new hip fracture SNF admissions from hospital are statistically significant at conventional levels when there is a >1% difference between the groups. MA patients were more likely to be married, black, Hispanic, and dual eligible for both Medicare and Medicaid; were less cognitively impaired upon SNF admission; and were more likely to have been treated in safety-net hospitals than FFS patients (Table 1). After applying IPTW, demographic and clinical characteristics of FFS and MA patients were very similar on most parameters. The main remaining differences were that MA patients were more likely to be admitted to for-profit chain SNFs compared to FFS patients. Calculating standardized differences between covariates after using IPTW revealed MA and FFS patients to be quite comparable (S1 Fig). Further evidence of clinical comparability after IPTW was demonstrated by no differences in all-cause 6-month and 1-year mortality between MA and FFS patients (S1 Table).

**Table 1:** Characteristics of FFS versus MA patients with hip fracture before and after inverse probability of treatment weighting.

Characteristic	Unadjusted		IPTW-adjusted	
	FFS, *n =* 211,296 (73.6%)	MA, *n =* 75,554 (26.3%)	FFS	MA
**Age (years)**	84.2 (7.5)	83.2 (7.5)	83.9 (8.8)	83.9 (14.5)
**Female**	77.4	76.5	77.2	77.3
**Married**	33.3	36.3	34.1	34.5
**Race/ethnicity**				
White	92.1	88.3	91.1	91.0
Black	2.9	4.1	3.2	3.2
Hispanic	3.0	5.8	3.7	3.6
Others	1.9	1.7	1.8	1.9
Dual	14.8	16.7	15.5	15.5
**Safety-net hospital**	22.3	25.5	23.1	23.1
**Hospital length of stay (days)**	4.9 (2.1)	5.0 (2.2)	5.0 (2.5)	4.9 (4.4)
**Intensive care length of stay (days)**	0.4 (1.5)	0.4 (1.5)	0.4 (1.7)	0.4 (2.9)
**HCC (comorbidity index)**	1.4 (0.5)	1.4 (0.5)	1.4 (0.6)	1.4 (1.1)
**Hospital acquired conditions**	18.6	17.3	18.2	18.5
**Fracture treatment**				
Open reduction internal fixation	24.3	25.6	24.7	24.6
Closed reduction internal fixation	20.8	21.5	21.0	21.0
Internal fixation	6.3	5.9	6.2	6.1
Partial hip replacement	27.8	28.1	27.8	27.9
Total hip replacement	2.7	3.1	2.8	2.8
Non-surgical management	17.8	15.6	17.3	17.3
**Body mass index (kg/m**^**2**^**)**	24.5 (5.0)	24.8 (5.1)	25.5 (5.9)	24.5 (9.8)
**Admission MDS-ADL score**	18.5 (3.2)	18.2 (3.2)	18.4 (3.8)	18.4 (6.1)
**Admission pain status**	45.6	44.9	45.7	44.6
**Cognition**				
Intact	54.8	59.7	56.1	55.9
Mild impairment	21.8	20.8	21.5	21.5
Moderate impairment	19.8	16.7	18.9	19.1
Severe impairment	3.5	2.6	3.3	3.3
**SNF characteristics and staffing**				
For profit	66.0	69.1	65.7	70.2
Part of chain	56.3	60.7	56.1	61.6
Total RN/LPN-FTE	26.7 (12.2)	28.3 (11.9)	26.6 (14.2)	28.3 (23.3)
Total PT-FTE	2.8 (3.5)	2.6 (3.3)	2.8 (4.0)	2.6 (6.9)
Total OT-FTE	2.6 (4.6)	2.5 (5.1)	2.6 (5.5)	2.5 (9.4)
Total MD-FTE	0.5 (1.0)	0.5 (0.9)	0.5 (1.2)	0.5 (1.8)

Data are given as mean (SD) or percent. Cognition categories: Measured by the Cognitive Function Scale (CFS) using the Cognitive Performance Scale (CPS) and Brief Interview for Mental Status (BIMS) from the Minimum Data Set admission assessment [23]. Admission pain status: If patient had pain that affected sleep and functional activity in last 5 days. Admission MDS-ADL score ranges from 0 to 28 (higher score indicates more impairment). Full-time equivalent (FTE): 35 hours/week of work in the SNF as staff or on contract.

FFS, Medicare fee-for-service; HCC, Hierarchical Condition Category; IPTW, inverse probability of treatment weighting; MA, Medicare Advantage; SNF, skilled nursing facility; total MD-FTE, total number of full-time equivalent physicians; total OT-FTE, total number of full-time equivalent occupational therapists; total PT-FTE, total number of full-time equivalent physical therapists; total RN/LPN FTE, total number of full-time equivalent registered nurses and licensed practical nurses.

Table 2 reveals that the average FFS patient spent 44.7 days (SD 41.7) in SNFs while MA patients spent only 36.9 days (SD 37.9) in these facilities. After adjusting for risk factors using the IPTW-weighted SNF fixed effects regression models (Table 2), MA patients spent 5.1 fewer days (95% CI -5.4 to -4.8) in SNFs than similar FFS patients. MA patients received 12.1 fewer minutes of rehabilitation therapy per day (95% CI -12.7 to -11.4) compared to FFS patients, and since FFS patients had longer SNF stays, they received 462.8 more minutes of total rehabilitation therapy than did MA patients in up to the first 40 days (95% CI -483.2 to -442.4).

**Table 2:** SNF length of stay and amount of rehabilitation care in FFS versus MA patients.

Outcome	Unadjusted			Adjusted	
	FFS, mean (SD) [median]	MA, mean (SD) [median]	Difference based on linear probability model (95% CI) [*p*-value]	Difference after IPTW-adjusted based on linear probability model (95% CI) [*p*-value]	Difference after IPTW-adjusted SNF fixed effects (95% CI) [*p*-value]
**SNF length of stay (days)**	44.7 (41.7) [31]	36.9 (37.9) [25]	−7.8 (−8.1 to −7.5) [<0.001]	−5.7 (−6.0 to −5.4) [<0.001]	−5.1 (−5.4 to −4.8) [<0.001]
**Rehabilitation therapy (minutes)**					
Total physical therapy	1,307.3 (614.1) [1,323.7]	1,003.9 (595.0) [946.2]	−303.3 (−316.3 to −290.4) [<0.001]	−279.2 (−283.8 to −274.7) [<0.001]	−241.9 (−252.7 to −231.1) [<0.001]
Total occupational therapy	1,159.3 (567.6) [1,169.9]	898.4 (553.7) [839.5]	−260.9 (−272.9 to −248.9) [<0.001]	−242.3 (−246.5 to −238.1) [<0.001]	−220.9 (−230.8 to −210.9) [<0.001]
Total rehabilitation therapy	2,466.7 (1,133.9) [2,519.2]	1,902.3 (1,106.8) [1,791.4]	−564.3 (−588.4 to −540.2) [<0.001]	−521.5 (−530.0 to −513.2) [<0.001]	−462.8 (−483.2 to −442.4) [<0.001]
Rehabilitation therapy/day	85.1 (22.9) [85.8]	71.3 (29.9) [74.6]	−13.8 (−14.5 to −13.0) [<0.001]	−13.7 (−13.9 to −13.5) [<0.001]	−12.1 (−12.7 to −11.4) [<0.001]

SNF length of stay: Follow-up to 180 days. Total therapy: Sum of therapy minutes (independent + concurrent + group) administered to the resident up to 40 days. Total rehabilitation therapy: Combined occupational therapy + physical therapy minutes. Rehabilitation therapy/day: Total rehabilitation therapy divided by length of stay up to 40 days. The 95% CIs and *p*-values are based on errors clustered by SNF.

FFS, Medicare fee-for-service; IPTW, inverse probability of treatment weighting; MA, Medicare Advantage; SNF, skilled nursing facility.

Table 3 presents the outcome comparisons using a linear probability model with SNF fixed effects as well as the results of a logistic regression model without SNF fixed effects. Even after IPTW adjustment, MA patients were discharged with about one-third of a point difference in functional status improvement on the MDS-ADL scale (−0.4 points, 95% CI −0.5 to −0.3) as compared to FFS patients. However, MA patients had a hospitalization rate that was more than 1 absolute percentage point lower than that of FFS patients (−1.2%, 95% CI −1.5 to −1.1). This translates into a relative difference of 16% based upon the logistic regression model (odds ratio 0.84, 95% CI 0.81–0.87). Moreover, MA patients were fully 3 percentage points more likely to have been successfully discharged to the community from the SNF (3.2%, 95% CI 2.7% to 3.7%) compared to FFS patients (Table 3), or 18% (odds ratio 1.18, 95% CI 1.15–1.20) more likely to have been successfully discharged.

**Table 3:** Patient outcomes in FFS versus MA patients before and after IPTW and SNF fixed effects.

Outcome	Unadjusted				Adjusted		
	FFS	MA	Difference based on linear probability model (95% CI) [*p*-value]	Odds ratio based on logit model (95% CI) [*p-*value]	Difference after IPTW-adjusted based on linear probability model (95% CI) [*p-*value]	Odds ratio based on logit model (95% CI) [*p*-value]	Difference after IPTW-adjusted SNF fixed effects (95% CI) [*p*-value]
Change in MDS-ADL score	3.7	3.2	−0.6 (−0.7 to −0.6) [<0.001]	NA	−0.7 (−0.8 to −0.6) [<0.001]	NA	−0.4 (−0.5 to −0.3) [<0.001]
30-day hospital readmission (%)	10.3	8.3	−1.9 (−2.1 to −1.6) [<0.001]	0.81 (0.78 to 0.83) [<0.001]	−1.3 (−1.0 to −1.5) [<0.001]	0.84 (0.81 to 0.87) [<0.001]	−1.2 (−1.5 to −1.1) [<0.001]
Became long-stay resident (%)	6.8	5.3	−1.5 (−1.7 to −1.3) [<0.001]	0.76 (0.73 to 0.79) [<0.001]	−0.7 (−0.9 to −0.4) [<0.001]	0.88 (0.84 to 0.92) [<0.001]	−0.6 (−0.8 to −0.3) [<0.001]
Successful discharge to community (%)	71.7	77.3	5.6 (5.2 to 6.0) [<0.001]	1.32 (1.29 to 1.35) [<0.001]	3.0 (2.6 to 3.4) [<0.001]	1.18 (1.15 to 1.20) [<0.001]	3.2 (2.7 to 3.7) [<0.001]

Change in MDS-ADL score: Discharge MDS-ADL score minus admission MDS-ADL score; the score was reversed (negative to positive) for better understanding. Higher score change indicates greater improvement in functional status. Long-stay resident: Stayed more than 100 days. Successful discharge to the community: Discharge to community within 100 days of SNF admission followed by uninterrupted 30 days’ stay in community/home/home health. The 95% CIs and *p*-values are based on errors clustered by SNF.

FFS, Medicare fee-for-service; IPTW, inverse probability of treatment weighting; MA, Medicare Advantage; NA, not applicable; SNF, skilled nursing facility.

Approximately 3.5% of the patients in our analytical cohort had severe cognitive impairment, which may differentially affect care processes and patient outcomes. Sensitivity analyses conducted after excluding patients with severe cognitive impairment revealed no change in the pattern of results (S2 and S3 Tables).

## Discussion

Comparing MA and FFS community-dwelling patients who had not had a prior nursing home stay or hospitalization in the past year who were hospitalized for hip fracture and transferred to SNFs, we found that MA patients had shorter SNF stays and received less rehabilitation care, but their outcomes were similar to, or better than, those of comparable FFS patients. Thus, 5 fewer days of SNF care and over 400 fewer minutes of therapy in the SNF did not appear to adversely affect Medicare beneficiaries’ ability to successfully transition home. These results control for numerous observed patient and facility characteristics, suggesting that it is possible to reduce SNF length of stay and achieve outcomes comparable to or better than those among FFS beneficiaries discharged from hospitals to SNFs following a hip fracture.

Our findings are consistent with a recent study revealing that, despite use of low-intensity care in post-acute settings (SNFs versus inpatient rehabilitation facilities), MA patients with lower extremity joint replacement, stroke, or heart failure manifested better outcomes than similar FFS patients [4]. Our study results are also consistent with prior work suggesting that the effect of additional therapy diminishes as the RUG level increases and that additional therapy after a certain threshold does not directly translate into greater likelihood of community discharge [26]. Indeed, our results expand upon these earlier findings by using a nationally representative, homogenous population of newly admitted acute care patients with hip fracture using in-depth information on the exact amount of PT and OT received. We chose hip fracture because it is an incident event that almost universally results in acute hospitalization, followed by discharge to a SNF [8]. The incident nature of the condition minimizes the “sample selection bias” of healthier patients electing to enroll in MA plans, and relatively sicker patients in FFS plans. Furthermore, including only patients with no prior hospitalization or SNF use in the last 12 months further reduces differences in case mix between MA and FFS patients. While we did find that MA patients were discharged with slightly less functional gain compared to FFS patients, a one-third point difference on the 28-point MDS-ADL scale is not generally considered clinically meaningful. Based on prior studies and clinicians’ opinion, a positive change of 2 points or more can be interpreted as a clinically meaningful gain in functional independence [18].

CMS and the Institute of Medicine have prioritized efforts to improve the value of post-acute care in SNFs, since post-acute care accounts for nearly 75% of the geographic variation in Medicare spending, and SNFs constitute the largest component of post-acute care spending in the US [27]. Our observation of shorter lengths of stay among MA compared to FFS patients, who are otherwise very similar and served by the same facility, can be viewed as informing ongoing efforts to develop value-based purchasing models focused on curtailing unnecessary utilization of SNF services, particularly since Medicare’s FFS payment rates for SNF care are 23% higher than MA rates [7].

While counterintuitive, our finding that MA patients are achieving superior outcomes in shorter periods and with less therapy has a plausible explanation. One possible mechanism is that MA plans encourage the use of narrow networks of efficient SNFs. In its March 2017 report to Congress, the Medicare Payment Advisory Commission reported that SNFs with greater efficiency in terms of low costs per day and good quality of care for 3 years had lower readmission rates and higher community discharge rates, despite having shorter stays than average [7]. MA plans have the ability to develop tight networks and selectively contract with efficient providers to form referral patterns between hospitals and SNFs, a practice that has been shown to be effective in reducing readmission rates [28]. In addition, unlike FFS plans, MA plans can use care management tools, such as deploying case managers or nurse practitioners to monitor therapy time and functional status improvement for each patient. This makes it possible for MA plans to be actively involved in the discharge planning process. This aligns with prior findings from the Evercare Medicare managed care program (Medicare+ Choice HMO), which reported that integrated managed care for nursing home residents was associated with fewer preventable hospitalizations [29].

Our study has several limitations. First, we used IPTW to adjust for potential selection bias in MA versus FFS enrollment and then compared those types of patients within each facility using fixed effects regression models; however, these strategies can only adjust for observed differences between patients and for facility confounders. Nonetheless, we observed no differences in adjusted 6-month and 1-year mortality rates between the 2 populations, which suggests that they are clinically comparable. Second, our study did not examine differences in home health utilization after discharge from SNFs, which could have facilitated accelerated SNF discharge. This is an important area to explore in future research since it could provide further insight into patients’ longer-term outcomes. Lastly, our findings are limited to patients with hip fracture; results may not be generalizable to other conditions where extensive rehabilitation and longer follow-up are needed. Despite these limitations, we believe our approach minimizes important limitations by including a comprehensive set of variables from different data sources.

### Conclusion

Despite rapidly increasing MA enrollment, to date there has been limited information about the quality of care and post-acute clinical outcomes for frail patients covered by MA plans. MA patients had shorter SNF length of stay and fewer minutes of rehabilitation therapy after hip fracture, but experienced lower rates of hospital readmission and long-term institutionalization and higher rates of successful discharge to the community than did similar FFS patients. These findings reflect well on the approach MA plans adopt regarding shorter SNF stays but also have implications for clinical recommendations regarding the amount of therapy required. This could certainly inform the current debate on value-based payment reform in post-acute settings. Moving forward, improving the efficiency and quality of post-acute care by reducing unnecessarily long rehabilitation stays in costly settings and shifting therapy care towards home-based services may be a new norm in value-based care.

## Supporting information

S1 STROBE Checklist STROBE checklist. (DOC)

S1 Fig Sample description using standardized differences before and after applying IPTW. (TIF)

S1 Protocol Study protocol. (DOCX)

S1 Table Differences in mortality rates between FFS and MA patients. (DOCX)

S2 Table Length of stay and amount of rehabilitation care in FFS versus MA patients after excluding patients with severe cognitive impairment. (DOCX)

S3 Table Patient outcomes in FFS versus AM patients before and after IPTW and SNF fixed effects after excluding patients with severe cognitive impairment. (DOCX)

## Abbreviations

ADL: activities of daily living
CMS: Centers for Medicare and Medicaid Services
DSH: disproportionate share hospital
FFS: Medicare fee-for-service
HCC: Hierarchical Condition Category
HEDIS: Healthcare Effectiveness Data and Information Set
IPTW: inverse probability of treatment weighting
MA: Medicare Advantage
MBSF: Master Beneficiary Summary File
MDS: Minimum Data Set
MedPAR: Medicare Provider and Analysis Review
OT: occupational therapy
PT: physical therapy
RUG: resource utilization group
SNF: skilled nursing facility
