# Supplementary figures and images for: Comparing post-acute rehabilitation use, length of stay, and outcomes experienced by Medicare fee-for-service and Medicare Advantage beneficiaries with hip fracture in the United States: A secondary analysis of administrative data
Source: PLoS Med. 2018 Jun 26;15(6):e1002592. doi: 10.1371/journal.pmed.1002592 (PMC6019094; doi:10.1371/journal.pmed.1002592)

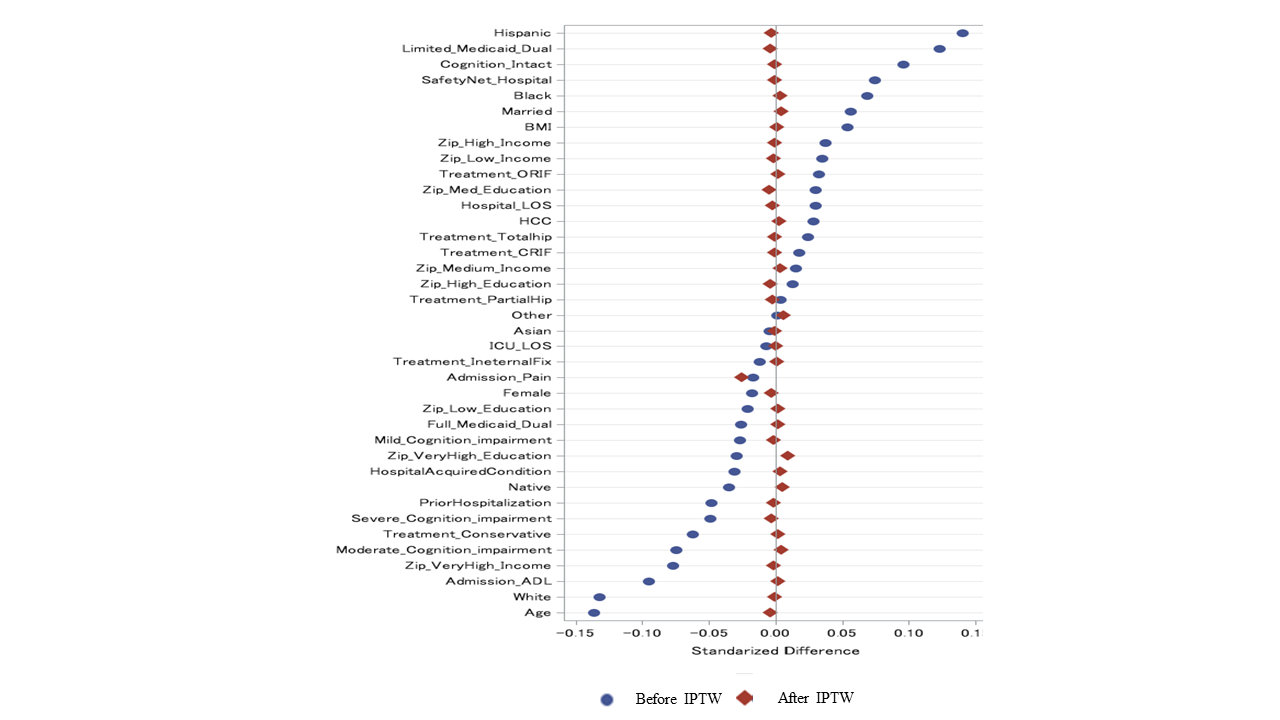

Supplement: S1 Fig — (TIF) [file pmed.1002592.s002.tif]
