# Supplementary material for: Comparing post-acute rehabilitation use, length of stay, and outcomes experienced by Medicare fee-for-service and Medicare Advantage beneficiaries with hip fracture in the United States: A secondary analysis of administrative data
Source: PLoS Med. 2018 Jun 26;15(6):e1002592. doi: 10.1371/journal.pmed.1002592 (PMC6019094; doi:10.1371/journal.pmed.1002592)
Supplement: S1 Protocol — (DOCX) [file pmed.1002592.s003.docx]

| Project Overview | |
| --- | --- |
| Original Project Title: | Comparing Length of Stay, Rehabilitation Services Utilization in Medicare Advantage versus Traditional Medicare Fee- For Service Beneficiaries with Hip Fracture in Skilled Nursing Facilities. |
| Principal Investigator | Vincent Mor |
| Lead Author | Amit Kumar |
| Coauthors | Momotazur Rahman, Amal Trivedi, Linda Resnik, Pedro Gozalo, Vincent Mor |
| Funding | This study was supported in part by National Institute of Aging grants P01AG027296, R01AG044374-01, R034G050002 |
| Data Usage Agreement | 18900 |
| Data Repository Link | <https://repository.library.brown.edu/studio/item/bdr:786344/> |
| Background | One in three Medicare enrollees received their insurance beneﬁts through Medicare Advantage (MA) plan.^1^ After the passage of the Medicare Modernization Act, there has been a rapid increase in MA enrollment and use of skilled nursing facilities (SNF) for post-acute rehabilitation services compared to inpatient rehabilitation facilities. Previous studies examining utilization of rehabilitation services and the quality of care have focused on Medicare fee-for-service (FFS) patients.^2^ However, there is no information about the rehabilitation quantity, quality, and outcomes of care among MA patients staying in SNF. The Office of Inspector General reported that SNFs upcode the FFS patients into higher payment RUGs categories, leading to more hours of therapy without a substantial rise in case-mix (age and diagnoses at admission were largely unchanged from 2006 to 2008).^3^ It is unknown whether this inappropriate selection of higher paying RUG therapy group in SNFs is affecting MA patients or not and how it influences the clinical outcome in these two groups. Our group has recently published a paper about rehabilitation service utilization in patients with hip fracture among Medicare FFS beneficiaries.^2^ Jung et al. reported that there is an increase in the quantity of therapy among FFS patients with hip fracture without change in case mix at SNF admission.^2^ Dr. Kumar is interested in extending this research in the MA population and comparing this with traditional FFS patients. Initially, we thought of using MDS-3 assessment completed on all Medicare- or Medicaid-certified nursing home residents irrespective of their insurance status. However, after doing a comprehensive literature review, we found that hospitals receiving a disproportionate share and educational training payments and supplements submit claims for MA beneficiaries.^4^ Therefore, we decided to use inpatient claims and HEEDIS for retrieving hospital related information. |

| **Data Sources** | **Variables** | **Link** | **Years** |
| --- | --- | --- | --- |
| Medicare Provider and Analysis Review (MedPAR) | Types of Fracture, Hospital length of stay, ICU, HCC score, hospital acquired conditions, and fracture management using ICD-9 procedure codes. | <https://www.resdac.org/cms-data/files/medpar-rif> | 2010-2015  Year 2010 was used to look back |
| Minimum Data Set (MDS 3.0) | Admission and Discharge ADL, Pain Status, Cognition, Pain, BMI | <https://www.resdac.org/cms-data/files/mds-3.0> | 2010-2015 |
| Master Beneficiary Summary File (MBSF) | Age, Sex, Race, Marital Status, Date of death, Enrollment Status: FFS, MA, dual eligibility | <https://www.resdac.org/cms-data/files/bsf> | 2011-2015 |
| Online Survey, Certification, and Reporting System (OSCAR) | SNF Characteristics, Profit Status, Part of Chain, Staffing Pattern: Total RN/LPN FTE, PT- FTE, OT-FTE, MD-FTE | <http://www.longtermcareinfo.com/data/casper-and-oscar.php> | 2011-2015 |
| Healthcare Effectiveness Data and Information Set (HEDIS) | Capture readmission for MA patients ‘if patients will readmit to non- DSH hospitals | <https://www.resdac.org/cms-data/files/hedis-puf> | 2011-2015 |
| American Community Survey | Zip code level median household income and education. | <https://www.census.gov/programs-surveys/acs/> | 2011-2015 |
| Residential History File (RHF) | Successful Discharge to the Community | RHF create a per-person chronological history of health service utilization and location of care within a pre-specified calendar using Medicare claims and post-acute assessment data.  <https://www.ncbi.nlm.nih.gov/pmc/articles/PMC3015013/> | 2011-2015 |

**Note:** These are person-level data (i.e., MDS, MBSF, MedPAR, and HEDIS) are covered under the strict terms of a Data Use Agreement (DUA) with the Centers for Medicare and Medicaid Services (CMS). We are prohibited from making any person-level data file, no matter how de-identified, available. However, researchers interested in replicating the results of these analyses may enter into their own DUA with CMS. Please contact the Research Data Assistance Center (ResDAC) at www.resdac.org for assistance. The OSCAR/CASPER is provider-level data, and zip-code level data from the American Community Survey are available for purchase/download. **Links are provided in the table above for more information.**

**Abbreviation:** FFS- Fee-for-Service; MA- Medicare Advantage; SNF- Skilled Nursing Facilities; HCC- Hierarchical Condition Category; ICU- Intensive Care Unit; ICD-9- International Classification of Diseases, Ninth Revision, Clinical Modification (**ICD**-**9**-CM);

ADL- Activities of Daily Living; BMI- Body Mass Index; Total RN/LPN FTE- Total number of full-time equivalent Registered Nurse and Licensed Practical Nurse; Total PT- FTE- Total number of full-time equivalent Physical Therapist; Total OT- FTE- Total number of full-time equivalent Occupational Therapist; Total MD- FTE- Total number of Full-time Physician;

DSH- Disproportionate Share Hospital,

**Timeline**:

| **Date** | **Proposed Activities** |
| --- | --- |
| November 2016 | First meeting to discuss research question- Amit Kumar, a postdoc fellow, presented his research ideas and hypothesis in our regular research meeting. |
| December 2016 | Comphrensive literature review |
| January 2017 | Presented research strategy with literature review |
| February 2017 | Data retrieval and proposal drafted |
| February 2017 | Analytical cohort created |
| March 2017 | Descriptive Analysis completed |
| March 2017 | Discuss results with clinician and coauthors |
| April 2017 | Try to develop instrumental variable but could not able to find good one |
| April 2017 | Propensity Score Matching and then decided to use IPTW |
| May 2017 | Presented final analysis after using IPTW weights and SNF fixed effects |
| May 2017 | Present the results in Annual PO advisory board meeting |
| June 2017 | Sensitivity Analysis to compare long term risk: 6 moths and 1year mortality |
| July 2017 | First draft of manuscript including all tables and figures |

| **Study Retrieval Request** | |
| --- | --- |
| Data Requested | November 14 2016 |
| Analytical Cohort Completed | February 2017 |
| Unit of Analysis | Individual level |
| Research Questions | 1. Evaluate the differences in post-acute rehabilitation services utilization (physical therapy, occupational therapy) in older patients enrolled in Medicare health maintenance organizations (HMOs) and fee-for-service (FFS) systems during a short stay in skilled nursing facilities (SNFs). 2. Compare the differences in patients centered outcome associated with amount of rehabilitation services utilization during a short stay in skilled nursing facilities (SNFs). |
| Working Hypothesis | FFS patients will be receiving more rehabilitation therapy and staying longer in the SNF, and will have better outcomes. |
| Study Sample | We will select patients with a hip fracture for this study because this is an acute condition that commonly occurs in frail older adults and majority if them receives rehabilitation care in the SNF. |
| **Inclusion Criteria** | **Dataset: MedPAR Year:** January 2010 (lookback period for 2011) through June 2015  Patients admitted to hospital with hip fracture  MS-DRG codes: 533, 534, 535, and 536  Primary ICD-9-CM diagnostic codes: 82000, 82021, 82022, 82023, 82024, 82026, 82027, 82028, 82029, 82030, 82031, 82032, 8208, 82080, 82009, and 8080  Age ≥ 65 years  Patients from hospital with no information on disproportionate share  Length of stay less than 15 days  Discharged to only SNF  Admitted to SNF within 3 days of discharge from hospital  Merge with American community Survey  Exclude previous nursing home stay in past twelve months  Exclude previous hospitalization in past twelve months  **Dataset: MDS-3** - **Inclusion Criteria**  First-time SNF admissions from acute care hospitals identified on the basis of the presence of an MDS admission assessment tracking record  Include short term and long-term stay  Patients must have at least two assessments.  Patients must have first MDS assessment within 10 days of admission to SNF  Patients must have at least second or subsequent MDS (last) assessment within 10 days of discharge from SNF or any assessment.  Exclude Patients in coma  Exclude patients’ with any missing information on variables  Exclude patients’ with extreme therapy minutes and outliers  Exclude patients’ from Hawaii, Virgin Island, and Puerto Rico  Since 2008, the MedPAR file contains claims information on MA enrollees receiving care in disproportionate share hospitals (DSH) and Indirect Medical Education or Direct Medical Education adjustments. Therefore, our study cohort will be limited to patients discharged from these hospitals. |
| **Primary Independent Variable** | **Physical therapy (PT) and Occupational Therapy (OT)**  OT/PT Individual Minutes Number: The data in this column indicates the total number of minutes that physical therapy was administered to the resident in the last seven days.  OT/PT Concurrent Minutes Number: The data in this column indicates the total number of minutes that physical therapy was administered to the resident concurrently with one other resident in the last seven days.  OT/PT Group Minutes Number: The data in this column indicates the total number of minutes that physical therapy was administered to the resident as part of a group of residents in the last seven days.  **Measure of Rehabilitation Therapy:** We will have an average mean number of minutes of therapy per week, on the basis of the 7-day retrospective period and all assessments from the entire SNF stay. We will sum total number of individual minutes, concurrent minutes, and group minutes for PT and OT services across all MDS assessment records during patients’ entire SNF stay. Since most stays will be less than 40 days, we will calculate minutes of therapy received in the first 40 days of SNF stays. Further, we will compute average daily minutes per day by dividing the total number of therapy minutes from the SNF length of stay up to 40 days.  **SNF Length of stay** will be defined as calendar days from admission to discharge using MDS admission and discharge records. We will follow-up to 180 days in the SNF. |
| **Dependent Variables** | **Function:** Functional status will be measured using seven ADL items into a 28-point scale with 0 indicating independence and 28 indicating total dependence. Change in functional status will be computed by subtracting admission from discharge scores; negative scores will be reversed to be positive to improve understanding. The more positive score the greater the improvement.  **Hospital Readmission (yes/no):** We will use all-cause readmission to the hospital within 30 days following discharge from the index hospitalization. Because MA enrollees may be readmitted to a non-DSH hospital which may not submit claims to Medicare, so we will merge the MedPAR and HEDIS files to estimate the 30-day hospital readmission for MA enrollees.  **Long-stay resident (yes/no):** SNF patient will become a long-stay nursing home resident, if they will stay in a nursing home more than 100 days.  **Successful Discharge to the community (yes/no):** Successful discharge from SNF will be defined as being discharged to the community within 100 days of SNF admission and remaining alive in the community without being institutionalized in any acute and post-acute settings for at least 30 days. We will link MDS with MedPAR and HEDIS files to estimate the Successful discharge to the community. |

**Original Statistical Analysis**

There are some evidence showing cherry picking of healthier beneficiaries in MA plans and sicker patients switching from MA to FFS. There might be a level of endogeneity in the selection of MA enrollees. We anticipated that MA and FFS beneficiaries might differ with respect to demographic, clinical, and socioeconomic characteristics. To account for differences in observed demographic and clinical characteristics between FFS and MA, we will generate a propensity score that will reflect the probability of individuals to be in MA versus FFS program using observable baseline characteristics. Propensity score model will include age, gender, race, marital status, length of stay in the hospital, number of days in the intensive care unit, fracture treatment, number of comorbidities, admission cognition, body mass index, hospital-acquired complications, HCC score, dual Medicare/Medicaid status, median household income, household education and state fixed effects. After that, Inverse Probability of Treatment Weighs (IPTW) will be generated from the propensity scores to balance the differences in patient demographic and clinical characteristics.

We will compare baseline characteristics between FFS and MA using chi-square tests for categorical variables and t-tests for continuous variables then weights were applied to calculate weighted rates and mean. First, we will use linear probability model without applying IPTW to compare the difference in outcomes between MA and FFS and then we will use IPTW.

After that, we will use a linear probability model to compare outcomes between FFS and MA patients after applying IPTW and SNF fixed-effect.

**After reviewer feedback:** We added odds ratio from logistic regression models to compare the binary outcome variables for 30-day hospital readmission, becoming a long-stay resident and successful discharge to the community.

**Recommended Tables**

**Table 1.** Characteristics of Medicare Fee-For-Service versus Medicare Advantage Patients with Hip Fracture before and after Inverse Probability of Treatment Weighting.

| **Variables** | **Unadjusted** | | **IPTW-adjusted** | |
| --- | --- | --- | --- | --- |
|  | FFS | MA | FFS | MA |
| Age |  |  |  |  |
| Female |  |  |  |  |
| Married |  |  |  |  |
| Race | | | | |
| White |  |  |  |  |
| Black |  |  |  |  |
| Hispanic |  |  |  |  |
| Others |  |  |  |  |
| Dual |  |  |  |  |
| Safety-net Hospitals % |  |  |  |  |
| Hospital Length of Stay |  |  |  |  |
| Intensive Care Length of Stay |  |  |  |  |
| HCC Score (Comorbidity Index) |  |  |  |  |
| Hospital Acquired Conditions % |  |  |  |  |
| **Fracture Treatment** | | | | |
| Open Reduction Internal Fixation |  |  |  |  |
| Close Reduction Internal Fixation |  |  |  |  |
| Internal Fixation |  |  |  |  |
| Partial Hip Replacement |  |  |  |  |
| Total Hip Replacement |  |  |  |  |
| Non-Surgical Management |  |  |  |  |
| Body Mass Index |  |  |  |  |
| Admission ADL |  |  |  |  |
| Admission Pain Status |  |  |  |  |
| **Cognition** | | | | |
| Intact |  |  |  |  |
| Mild Impairment |  |  |  |  |
| Moderate Impairment |  |  |  |  |
| Severe Impairment |  |  |  |  |
| **SNF Characteristics % and Staffing (mean ± SD)** | | | | |
| For Profit |  |  |  |  |
| Part of Chain |  |  |  |  |
| Total RN/LPN FTE |  |  |  |  |
| Total PT- FTE |  |  |  |  |
| Total OT-FTE |  |  |  |  |
| Total MD-FTE |  |  |  |  |

Cognition categories: Measured by the Cognitive Function Scale (CFS) using Cognitive Performance Scale (CPS) and Brief Interview for Mental Status (BIMS) from MDS admission assessment. Pain categories: If patient is having pain that affects sleep and functional activity in last 5 days Admission ADL score ranges from 0 - 28 (higher scores indicate more impairment). Full-time equivalent (FTE): 35 hours’/ week work in the SNF as a staff or on contract.

**Abbreviation:** FFS- Fee-for-Service; MA- Medicare Advantage; SNF- Skilled Nursing Facilities; HCC- Hierarchical Condition Category; ADL- Activities of Daily Living; SD- Standard Deviation; IPTW- Inverse Probability of Treatment Weighting; FTE- Full-Time Equivalent; Total RN/LPN FTE- Total number of full-time equivalent Registered Nurse and Licensed Practical Nurse; Total PT- FTE- Total number of full-time equivalent Physical Therapist; Total OT- FTE- Total number of full-time equivalent Occupational Therapist; Total MD- FTE- Total number of Full-time Physician.

**p<0.01, *p<0.05

**Table 2**: Length of Stay and Amount of Rehabilitation Care in Medicare Fee-For-Service versus Medicare Advantage Patients

|  | **Unadjusted** | | | **Adjusted** | |
| --- | --- | --- | --- | --- | --- |
|  | FFS | MA | Differences based on linear probability model  (95% CI)  [p-value] | Differences after IPTW-Adjusted based on linear probability model  (95% CI)  [p-value] | Differences after IPTW-Adjusted SNF Fixed Effect (95% CI)  [p-value] |
| SNF Length of Stay  Mean (SD) [median] |  |  |  |  |  |
| Rehabilitation Therapy (Minutes) Mean (SD) [median] | | | | | |
| Total Physical Therapy |  |  |  |  |  |
| Total Occupational Therapy |  |  |  |  |  |
| Total Rehabilitation Therapy |  |  |  |  |  |
| Rehabilitation Therapy/Day |  |  |  |  |  |

**Note:** SNF Length of Stay: follow-up to 180 days. Total Therapy: Sum of therapy minutes (Independent + Concurrent + Group) administered to the resident up to 40 days. Total Rehabilitation Therapy: Combined Occupational therapy + Physical therapy minutes. Rehabilitation Therapy/day: Total Rehabilitation therapy divided by length of stay up to 40 days.

**Table 3:** Patients Outcomes in Medicare Fee-For-Service versus Medicare Advantage Patients before and after Inverse Probability of Treatment Weighting and SNF Fixed Effect.

|  | **Unadjusted** | | | | **Adjusted** | | |
| --- | --- | --- | --- | --- | --- | --- | --- |
|  | FFS | MA | Differences based on linear probability model  (95% CI)  [p-value] | *Odds Ratio based on logit model  (95% CI)  [p-value] | Differences after IPTW Adjusted  based on linear probability model  (95% CI)  [p-value] | *Odds Ratio based on logit model  (95% CI)  [p-value] | Differences after IPTW-Adjusted SNF Fixed Effect  [p-value] |
| Change in ADL |  |  |  |  |  |  |  |
| 30-Day Hospital Readmission % |  |  |  |  |  |  |  |
| Became Long-Stay Resident % |  |  |  |  |  |  |  |
| Successful Discharge to Community % |  |  |  |  |  |  |  |

**Notes:** Change in ADL: (Discharge ADL - Admission ADL) and the score was reversed in positive for better understanding. Higher score in ADL change indicates greater improvement in functional status. Long Stay Resident: Stayed more than 100 days. Successful

Discharge to the Community: Discharge to community within 100 days in SNF followed by uninterrupted 30 days stay in Community/home/home health.

*Odds Ratio based on logit model (95% CI) [p-value] was added after reviewer feedback

**References**

1. Gretchen Jacobson, Anthony Damico, Tricia Neuman, Gold M. Medicare Advantage 2017 Spotlight: Enrollment Market Update. 2017; <http://files.kff.org/attachment/Issue-Brief-Medicare-Advantage-2017-Spotlight-Enrollment-Market-Update>. Accessed July1, 2017.

2. Jung H-Y, Trivedi AN, Grabowski DC, Mor V. Does More Therapy in Skilled Nursing Facilities Lead to Better Outcomes in Patients With Hip Fracture? *Physical Therapy.* 2016;96(1):81-89.

3. Department of Health and Human Services. Inappropriate payments to Skilled Nursing Facilities cost Medicare more than a billion dollars in 2009. 2009; <https://oig.hhs.gov/oei/reports/oei-02-09-00200.pdf>. Accessed April 3, 2017.

4. Huckfeldt PJ, Escarce JJ, Rabideau B, Karaca-Mandic P, Sood N. Less Intense Postacute Care, Better Outcomes For Enrollees In Medicare Advantage Than Those In Fee-For-Service. *Health affairs (Project Hope).* 2017;36(1):91-100.
