# Supplementary material for: Comparing post-acute rehabilitation use, length of stay, and outcomes experienced by Medicare fee-for-service and Medicare Advantage beneficiaries with hip fracture in the United States: A secondary analysis of administrative data
Source: PLoS Med. 2018 Jun 26;15(6):e1002592. doi: 10.1371/journal.pmed.1002592 (PMC6019094; doi:10.1371/journal.pmed.1002592)
Supplement: S1 Table — (DOCX) [file pmed.1002592.s004.docx]

**S1 Table:** Differences in mortality rates between FFS and MA patients

|  | **Before IPTW** | | | | **IPTW-Adjusted** | |
| --- | --- | --- | --- | --- | --- | --- |
|  | **FFS**  **%** | **MA**  **%** | **Differences based on linear probability model**  **(95% CI)**  **[p-value]** | **Odds Ratio based on logit model**  **(95% CI)**  **[p-value]** | **Differences after IPTW-Adjusted based on linear probability model**  **(95% CI)**  **[p-value]** | **Odds Ratio based on logit model**  **(95% CI)**  **[p-value]** |
| Six-Month Mortality | 8.9 | 7.9 | -1.1  (-1.4 to -0.9)  [<.0001] | 0.88  (0.85 to 0.91)  [<.0001] | -0.1  (-0.3 to 0.3)  [0.502] | 1.01  (0.97 to 1.04)  [0.501] |
| **One-Year Mortality** | 9.8 | 8.9 | -1.1  (-1.0 to -0.9)  [<.0001] | 0.89  (0.86 to 0.92)  [<.0001] | -0.2  (-0.3 to 0.5)  [0.287] | 1.01  (0.98 to 1.05)  [0.285] |
