# Supplementary material for: Comparing post-acute rehabilitation use, length of stay, and outcomes experienced by Medicare fee-for-service and Medicare Advantage beneficiaries with hip fracture in the United States: A secondary analysis of administrative data
Source: PLoS Med. 2018 Jun 26;15(6):e1002592. doi: 10.1371/journal.pmed.1002592 (PMC6019094; doi:10.1371/journal.pmed.1002592)
Supplement: S2 Table — (DOCX) [file pmed.1002592.s005.docx]

**S2 Table:** Length of Stay and Amount of Rehabilitation Care in Medicare Fee-For-Service versus Medicare Advantage Patients after excluding patients with severe cognitive impairment**.**

|  | **Unadjusted** | | | **Adjusted** | |
| --- | --- | --- | --- | --- | --- |
|  | **FFS**  **Mean (SD) [median]** | **MA**  **Mean (SD) [median]** | **Differences based on linear probability model**  **(95% CI)**  **[p-value]** | **Differences after IPTW-Adjusted based on linear probability model**  **(95% CI)**  **[p-value]** | **Differences after IPTW-Adjusted SNF Fixed Effect (95% CI)**  **[p-value]** |
| SNF Length of Stay | 44.4  (41.0)  [31] | 36.5  (37.1)  [25] | -7.8  (-8.3 to -7.3)  [<.0001] | -5.7  (-6.2 to -5.2)  [<.0001] | -5.3  (-5.7 to -4.8)  [<.0001] |
| Rehabilitation Therapy (Minutes) | | | | | |
| Total Physical Therapy | 1320.2  (610.7)  [1337.8] | 1010.0  (594.1)  [951.4] | -310.2  (-323.3 to -297.0)  [<.0001] | -283.0  (-295.4 to -270.5)  [<.0001] | -246.3  (-257.3 to -235.3)  [<.0001] |
| Total Occupational Therapy | 1171.6  (563.9)  [1182.8] | 904.0  (552.7)  [845.2] | -267.5  (-279.7 to -255.3)  [<.0001] | -246.2  (-257.9 to -234.4)  [<.0001] | -225.5  (-235.6 to -215.4)  [<.0001] |
| Total Rehabilitation Therapy | 2491.9  (2554.2)  [1126.7] | 1914.1  (1800.2)  [1105.5] | -577.7  (-602.1 to -553.3)  [<.0001] | -529.2  (-552.6 to -505.8)  [<.0001] | -471.8  (-492.5 to 451.1)  [<.0001] |
| Rehabilitation Therapy/Day | 85.9  (22.3)  [86.6] | 71.8  (29.7)  [74.8] | -14.0  (-14.8 to -13.3)  [<.0001] | -13.8  (-14.5 to -13.1)  [<.0001] | -12.2  (-12.8 to -11.5)  [<.0001] |

Note: SNF Length of Stay: follow-up to 180 days. Total Therapy: Sum of therapy minutes (Independent + Concurrent + Group) administered to the resident up to 40 days. Total Rehabilitation Therapy: Combined Occupational therapy + Physical therapy minutes. Rehabilitation Therapy/day: Total Rehabilitation therapy divided by length of stay up to 40 days. The 95% CIs and p-values are based on errors clustered by SNF.
