# Supplementary material for: Comparing post-acute rehabilitation use, length of stay, and outcomes experienced by Medicare fee-for-service and Medicare Advantage beneficiaries with hip fracture in the United States: A secondary analysis of administrative data
Source: PLoS Med. 2018 Jun 26;15(6):e1002592. doi: 10.1371/journal.pmed.1002592 (PMC6019094; doi:10.1371/journal.pmed.1002592)
Supplement: S3 Table — (DOCX) [file pmed.1002592.s006.docx]

**S3 Table:** Patients Outcomes in Medicare Fee-For-Service versus Medicare Advantage Patients before and after Inverse Probability of Treatment Weighting and SNF Fixed Effect after excluding patients with severe cognitive impairment.

|  | **Unadjusted** | | | | **Adjusted** | | |
| --- | --- | --- | --- | --- | --- | --- | --- |
|  | **FFS** | **MA** | **Differences based on linear probability model**  **(95% CI)**  **[p-value]** | **Odds Ratio based on logit model**  **(95% CI)**  **[p-value]** | **Differences after IPTW-Adjusted**  **based on linear probability model**  **(95% CI)**  **[p-value]** | **Odds Ratio based on logit model**  **(95% CI)**  **[p-value]** | **Differences after IPTW-Adjusted SNF Fixed Effect**  **[p-value]** |
| Change in ADL | 3.6 | 3.0 | -0.6  (- 0.7 to -0.5)  [<.0001] | --- | -0.7  (-0.8 to -0.6)  [<.0001] | --- | -0.4  (- 0.5 to -0.4)  [<.0001] |
| 30-Day Hospital Readmission % | 10.0 | 8.1 | -1.5  (-1.8 to -1.3)  [<.0001] | 0.80  (0.78 to 0.83)  [<.0001] | -1.3  (-1.5 to -1.0)  [<.0001] | 0.83  (0.80 to 0.87)  [<.0001] | -1.5  (-1.8 to -1.2)  [<.0001] |
| Became Long-Stay Resident % | 7.4 | 5.7 | -1.6  (-1.9 to -1.4)  [<.0001] | 0.76  (0.73 to 0.79)  [<.0001] | -0.8  (-1.0 to -0.5)  [<.0001] | 0.87  (0.84 to 0.91)  [<.0001] | -0.7  (-1.0 to -0.5)  [<.0001] |
| Successful Discharge to Community % | 77.3 | 81.8 | 4.4  (4.1 to 4.8)  [<.0001] | 1.31  (1.28 to 1.35)  [<.0001] | 2.8  (2.4 to 3.2)  [<.0001] | 1.19  (1.16 to 1.22)  [<.0001] | 3.6  (3.1 to 4.0)  [<.0001] |

Notes: Change in ADL: (Discharge ADL - Admission ADL) and the score was reversed in positive for better understanding. Higher score in ADL change indicates greater improvement in functional status. Long Stay Resident: Stayed more than 100 days. Successful Discharge to the Community: Discharge to community within 100 days in SNF followed by uninterrupted 30 days stay in Community/home/home health. The 95% CIs and p-values are based on errors clustered by SNF.
